# A Rare Case Report on Holoprosencephaly With Cyclopia: Jimma University Medical Center, Ethiopia

**DOI:** 10.1155/crog/2602471

**Published:** 2026-03-07

**Authors:** Dejene Tolossa Debela, Fanta Asefa Disasa, Anberbir Girma, Sagni Jelkeba Seto

**Affiliations:** ^1^ Department of Obstetrics and Gynecology, Jimma University, Jimma, Ethiopia, ju.edu.et; ^2^ Department of Ophthalmology, Jimma University, Jimma, Ethiopia, ju.edu.et

**Keywords:** alobar, cyclopia, Ethiopia, holoprosencephaly, Jimma

## Abstract

**Background:**

Holoprosencephaly is a congenital brain malformation resulting from incomplete division of the forebrain (prosencephalon) into two hemispheres during early embryonic development, typically during the 4th–6th week of gestation. Cyclopia is a rare and fatal form of holoprosencephaly characterized by severe craniofacial abnormalities resulting from incomplete cleavage of the embryonic prosencephalon, leading to the failure of separation of the orbital cavities. The prevalence is 1.31 per 10,000 for live births and 1:250 for spontaneous abortions.

**Case Presentation:**

This is a case report of a 33‐year‐old Gravida 5, Para 4 woman with no history of abortion and a prior history of normal vaginal deliveries, who presented to the obstetric triage with a 7‐day history of absent fetal movements. After a detailed history was taken, a physical examination and ultrasound scan confirmed an intrauterine fetal death. Termination of the pregnancy was decided after the mother was counseled on the possible outcome of the pregnancy. Induction with misoprostol per the hospital protocol was given to deliver an 1800‐g, Grade 3 macerated male fetus with a diagnosis of a severe form of holoprosencephaly with cyclopia and proboscis.

**Conclusion:**

Although holoprosencephaly is rare, early first‐trimester anatomical ultrasound conducted by an experienced clinician is critical for accurate diagnosis, and evaluation of severity is pivotal for counseling families regarding expected outcomes and pregnancy termination decisions.

## 1. Introduction

Holoprosencephaly (HPE) is a developmental defect of the embryonic forebrain or prosencephalon, that is commonly associated with midfacial defects [1, 2]. The defect results from incomplete development of central nervous system (CNS) structures and has a spectrum of presentations [3, 4]. There are three classic types (from the most to least severe CNS defects): alobar, semilobar, and lobar [5].

The prevalence of cyclopia is 1 in 100,000 births globally. Although the exact etiology of HPE is not yet elucidated, the primary cause of the malformation is suspected to be heterogeneous, with both genetic (sonic hedgehog [SHH] and zinc finger protein of the Cerebellum 2 [ZIC2]) and environmental components involving teratogenic exposures, maternal diabetes, retinoic acid, and drug and alcohol abuse during early pregnancy [6].

## 2. Case Presentation

This is a case report of a 33‐year‐old Gravida 5, Para 4, claimed to be amenorrhic for the last 9 months and has no history of abortion. Her previous delivery history was a normal vaginal birth. She is currently presented with a complaint of absent fetal movement of 7 days duration to our hospital′s obstetric triage. For this pregnancy, she had two antenatal care (ANC) contacts at 2 and 6 months of amenorrhea at a nearby health center but was not scanned by obstetric ultrasound. She felt fetal quickening at 5 months of amenorrhea. She was referred from the health center with the diagnosis of third trimester pregnancy and IUFD for better investigation and management. The mother has no history of alcohol abuse, smoking, or any chronic medical illness throughout this pregnancy. After detailed history was taken, physical examination and ultrasound scanning confirmed an intrauterine fetal death. The obstetric ultrasound was done by a fetal–maternal medicine (MFM) fellow and the findings are as follows: single intrauterine pregnancy, negative fetal heartbeat, cephalic presentation with collapsed skull bone, and femoral length of 73 mm. The congenital anomaly was not detected antenatally due to the collapsed head and other structures.

All basic and other important laboratory investigations including random blood sugar, blood film, and complete blood count with differential were done and in normal range (please see Table 1).

**Table 1:** Details of all investigation laboratory of the mother.

Lists of investigations	Result of the tests	Remark
Random blood sugar (mg/dL)	101	
CBC		
WBC (c/uL)	11.1∗10^3^	
Hemoglobin (g/dL)	11.9	
Hematocrit (%)	34.9	
Platelet (c/uL)	390∗10^3/^	
VDRL	Negative	
HBSAg	Negative	
Urinalysis	Nonrevealing	
Blood group	O	
Rh status	Negative	
AST (U/L)	31	
ALT (U/L)	27	
PT (s)	13	
aPTT (s)	26	
INR (standardized)	1.1	

Abbreviations: %, percent; ALT, alanine aminotransferase; aPTT, activated partial thromboplastin time; AST, aspartate aminotransferase; CBC, complete blood count; c/uL, count per unilitre; g/dL, gram per decilitre; HBSAg, hepatitis B surface antigen; INR, international normalized ratio; mg/dL, milligram per decilitre; PT, prothrombin time; Rh, Rhesus; VDRL, venereal disease research laboratory; WBC, white blood cell.

**Figure 1:**
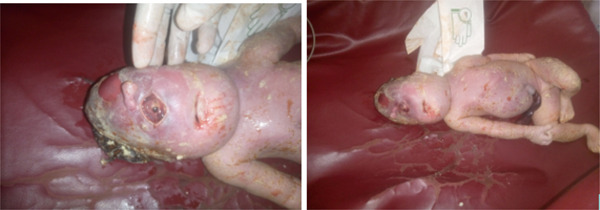
Dead fetus with single middle eye, proboscis above the eye, and macerated skin; (Explicit written consent to publish the clinical details and photographs was given by the family).

Termination of the pregnancy was decided after the mother was counseled by a multidiscipline team on the possible outcome and prognosis of the pregnancy. Induction with misoprostol as per the hospital′s protocol was done to deliver an 1800‐g Grade 3 macerated male fetus with a diagnosis of a severe form of HPE with cyclopia (see Figure 1).

The neonate was grossly examined postdelivery, and there was a single midline‐located eye with a proboscis in the midline above it and microcephaly with no well‐developed nasal aperture. Other associated defects were assessed but there is no cleft lip or palate. All other organs were grossly examined and were normal including the maxilla, umbilical cord, and the spine. Postmortem examination was not done due to the unavailability of service in our hospital.

## 3. Discussion

This rare case report is a case with a severe form of HPE with cyclopia diagnosed in the postnatal period in a newborn. Failure of a complete or partial division of the developing prosencephalon into hemispheres and lobes leads to this complex neural abnormality of HPE during the 4th–6th week of gestation. The exact cause of HPE is left unknown, but research revealed there are multiple risk factors including teratogenic exposures and genetic causes [6–8].

Evidence primarily from animal studies and mixed results from human epidemiological research showed possible risk factors including long use of aspirin, statins, poor glycemic control (hyperglycemia), methotrexate, and high alcohol consumption [9, 10]. Trisomies 13, 18, and 21 are the most common chromosomal disorders associated with HPE [11]. However, in this case report, the mother did not report an exposure to the above risk factors. Also, we ruled out TORCH infections in early pregnancy as they potentially affect the neurological embryogenesis leading to HPE defects [12, 13].

There were few reports in Ethiopia on similar cases with different clinical features and no identified causes or risk factors [13–15].

High index of suspicion is needed to diagnose HPE prenatally using obstetric ultrasound with the prominent clinical manifestations: absence of the eyes, cebocephaly (a flattened nose with a single nostril and closely spaced eyes [hypotelorism]), cyclopia, proboscis, and micrognathia (undersized mandible) [16]. Routine antenatal ultrasound can detect severe forms of HPE as early as the first trimester of pregnancy, which allows parents to receive timely and accurate counseling about the prognosis, potential associated conditions, and management options [17]. However, in our case, the diagnosis was not made due to lack of prenatal ultrasound examination. Cyclopia, proboscis, and cheilo/palatoschisis are associated with the incidence of severe form of HPE [18], which is also similar to our case.

## 4. Conclusion

Cyclopic HPE is a rare but severe form of HPE with a complex and multifactorial pathogenesis with poor prognosis. Early first‐trimester anatomical ultrasound conducted by an experienced clinician is critical for accurate diagnosis, with severity grading playing a central role in family counseling on prognosis and decisions about pregnancy termination. Further study is recommended to understand the possible risk factors and early diagnosis of HPE in Ethiopia.

## Author Contributions

D.T.D. and A.G. wrote the main manuscript, collected the data, and drafted the manuscript. F.A.D. and S.J.S. collected the data and prepared figures.

## Funding

No funding was received for this manuscript.

## Disclosure

All authors confirmed the final submitted manuscript.

## Ethics Statement

The authors declare that this work was done with all due respect to the code of ethics under the supervision of the Jimma University Institute of Health Institutional Review Board.

## Consent

Written informed consent was obtained from the patient for the images and potential publication of the case.

## Conflicts of Interest

The authors declare no conflicts of interest.

## Data Availability

The data that support the findings of this study are available from the corresponding author upon reasonable request.
